# Long-term clinical outcomes of [^177^Lu]Lu-DOTATATE in patients with metastatic neuroendocrine tumors

**DOI:** 10.3389/fonc.2024.1393317

**Published:** 2024-05-16

**Authors:** Sabah Iqbal, Eric Zhuang, Moses Raj, Nathan Bahary, Dulabh K. Monga

**Affiliations:** ^1^ Mercy Catholic Medical Center, Darby, PA, United States; ^2^ Department of Hematology and Oncology, Allegheny Health Network Cancer Institute, Pittsburgh, PA, United States

**Keywords:** gastroenteropancreatic neuroendocrine tumors, metastatic neuroendocrine tumors, [^177^Lu]Lu-DOTATATE, peptide receptor radionuclide therapy, [^68^Ga]Ga-DOTATATE

## Abstract

The incidence of gastroenteropancreatic neuroendocrine tumors has been rising and these tumors are usually only diagnosed at a metastatic stage. Present first line treatments include somatostatin analogs, targeted therapies and peptide receptor radionuclide therapy. The Lutetium-177 [^177^Lu] based radiotracer [^177^Lu]Lu-DOTATATE has only been approved as first-line treatment of metastatic midgut NETs however its efficacy as a third line or above treatment in patients with non ileal primaries has not been tested. In our study, we identified 25 patients with histologically confirmed well-differentiated metastatic neuroendocrine tumors and administered [^177^Lu]Lu-DOTATATE as a second line, third line and fourth line treatment. Our study demonstrated a notable response in patients with non-ileal primaries and heavily pretreated disease, warranting further studies for additional cycles of treatment.

## 1 Introduction

Neuroendocrine tumors (NET), also known as neuroendocrine neoplasms are epithelial neoplasms arising from neuroendocrine cells of various organs, with an incidence of 6-7 cases per 100,000 people in the United States. The most commonly involved sites are the lungs and the gastroenteropancreatic (GEP) system. In the past decade, owing to improvements in therapies, prolonged survival has been seen despite a steady rise in the incidence and prevalence of NETs (1, 2).

Based on histology, they are further classified into well-differentiated NETs and poorly-differentiated neuroendocrine carcinomas (NECs). GEP NETs are a heterogeneous group of neoplasms comprising well-differentiated NETs of the tubular gastrointestinal tract, previously known as carcinoid tumors.

**Figure 1:**
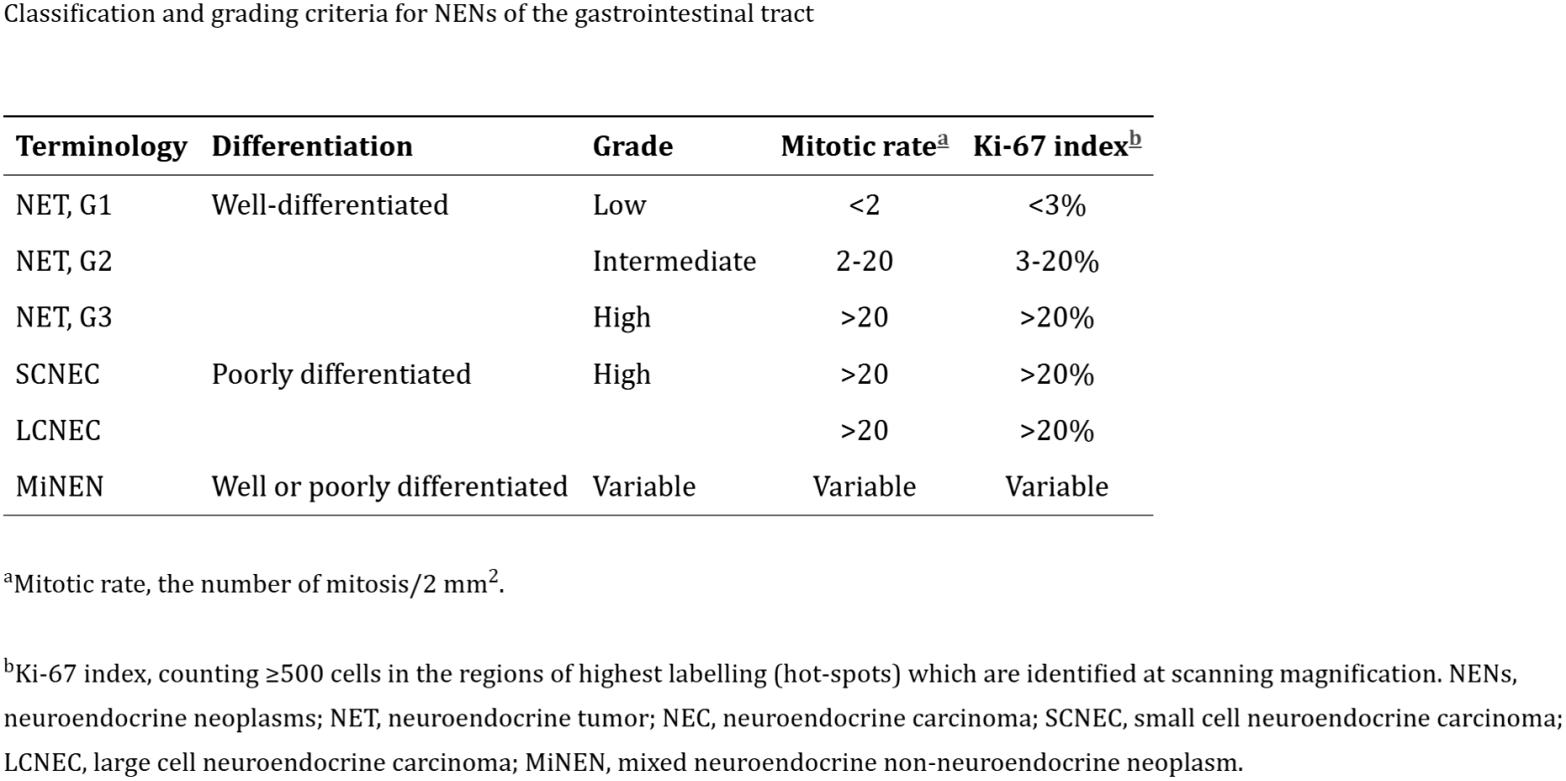
Classification and grading criteria for Neuroendocrine neoplasms (NEN) of the GI tract.

Based on the histologic appearance and proliferative rate, as assessed by mitotic count and Ki-67 labeling index, the World Health Organization (WHO) further classifies NETs as low grade (grade 1) and intermediate grade (grade 2) (3). The WHO also identifies a third category, the high-grade (grade 3) tumor, which is G2 by mitotic count but has a high proliferation rate. Poorly differentiated NECs are further classified as small-cell or large-cell type NECs and have a worse prognosis (**Figure 1**).

**Figure 2:**
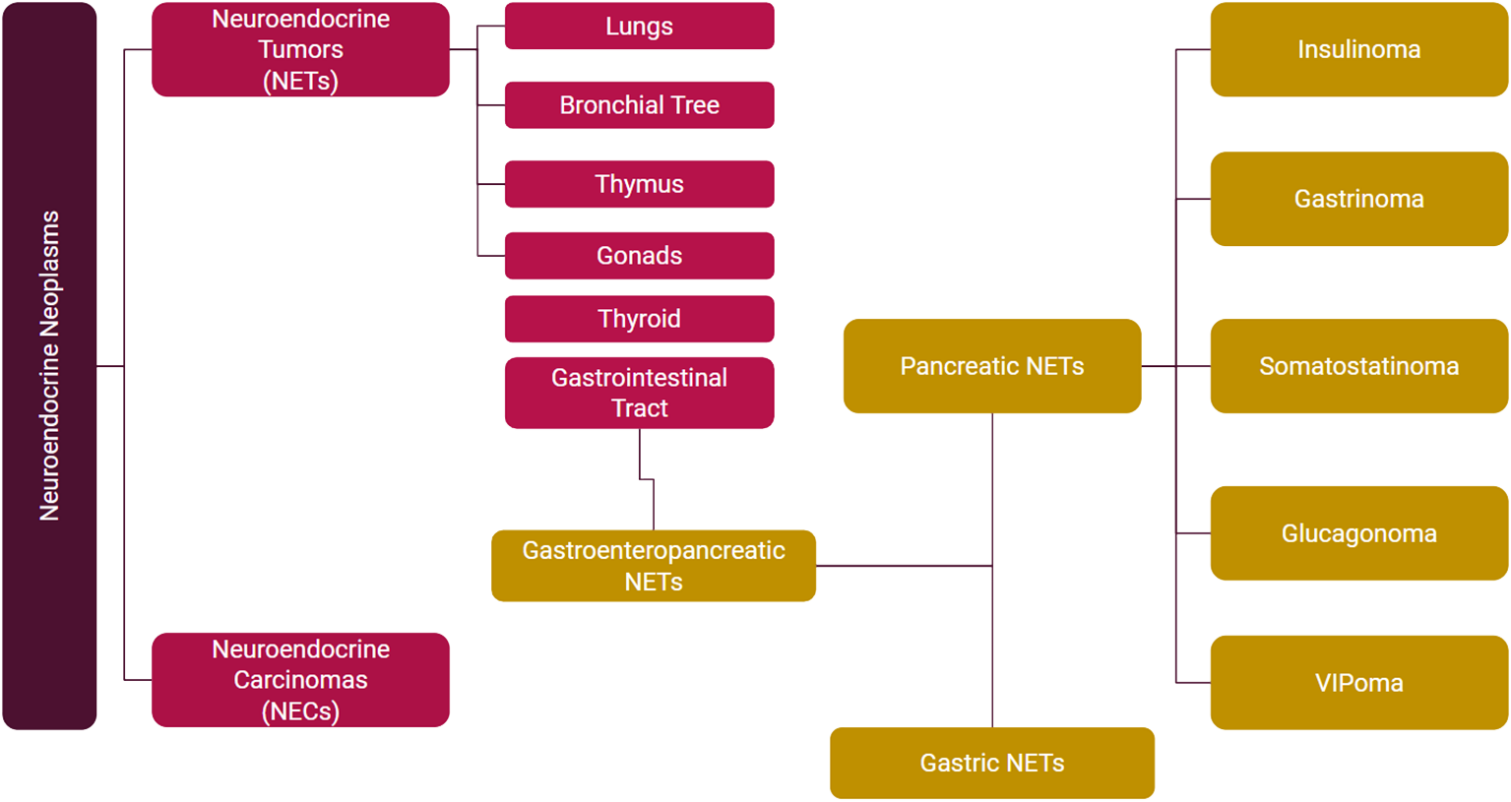
Clinical classification of NENs.

Based on hormone secretion, they are divided into functional and nonfunctional. Functional NETs are further characterized by the type of hormone secretion into insulinoma, gastrinoma, glucagonoma, VIPoma, and somatostatinoma (**Figure 2**). Midgut NETs typically metastasize to the liver and can present as carcinoid syndrome. Carcinoid syndrome can also be seen without liver metastases in patients with extraintestinal (e.g. ovarian, bronchial) NETs.

Most patients with GEP NETs remain symptom-free and hence remain underdiagnosed until the metastatic stage. They usually become symptomatic due to hormone hypersecretion rather than tumor bulk. Prognosis depends on multiple factors such as the primary tumor location with the worst prognosis seen in lung and colon primaries and tumors with high histologic grade or proliferative rate. The proliferative rate is an individual marker of prognosis irrespective of the grade (4, 5).

Multiphasic computed tomography (CT) or magnetic resonance imaging (MRI) is the imaging of choice for GEP NETs. For metastatic GEP NETs, a triphasic helical CT of the abdomen and pelvis is preferred.

All these tumors whether functional or nonfunctional often over-express somatostatin receptors (SSTR) with SSTR2 and SSTR5 subtypes being the most common. These receptors can be used as both diagnostic and therapeutic targets. One such technique is a Positron Emission Tomography (PET) based somatostatin receptor-based imaging technique. In 2016, US Food and Drug administration (FDA) approved gallium [^68^Ga]Ga-DOTATATE, as a routine imaging modality for NETs, which works by binding to these receptors with an affinity for SSRT2. It has a higher sensitivity and is preferred for small-volume and occult primary tumors (6). Other radionuclides that have been approved for similar imaging are gallium [^68^Ga]Ga-DOTATATE and copper [^64^Cu]Cu-DOTATATE (7, 8). These tests can also be used as a predictive tool for clinical response to therapy with somatostatin analogs.

In the absence of extrahepatic involvement, resection is the optimal treatment for hepatic metastases and in some cases, concomitant resection of the primary site has proven beneficial. In patients with GEP NETs with hepatic predominant disease, hepatic arterial embolization is an alternative, however survival benefits are undefined.

Recent studies in the last decade have identified first-line treatments for advanced diseases including somatostatin analogs (octreotide and lanreotide), targeted therapies (everolimus and sunitinib), and peptide receptor radionuclide therapy (PRRT).

An observational time and motion study in patients with GEP NETs where patients were treated with lanreotide or octreotide LAR showed that lanreotide was associated with a significant reduction in drug delivery time (2.5 minutes) as compared to octreotide LAR (6.2 minutes), contributing to an improvement in overall healthcare efficiency (9).

The ECOG-ACRIN E2211 study, a phase 2 trial compared the efficacy of temozolomide versus capecitabine/temozolomide in patients with advanced low-grade and intermediate-grade pancreatic NETs. Median PFS was 14.4 months for temozolomide versus 22.7 months for capecitabine/temozolomide (hazard ratio 0.53, p 0.022) in the median OS was 53.8 months for temozolomide and 58.7 months for capecitabine/temozolomide (hazard ratio 0.82, p 0.42). Median PFS and RR observed in the group that received combined treatment was the highest reported in a randomized study for pancreatic NETs (10).

Everolimus, an oral inhibitor of the mammalian target of rapamycin (mTOR), and sunitinib, a multi-targeted tyrosine kinase inhibitor, have both shown promising results in pancreatic NETs (11, 12). However, no significant difference in overall survival was established.

In that regard, [^177^Lu]Lu-DOTATATE has been identified as a promising first-line treatment in metastatic midgut NETs, with a better safety profile (13). PRRT (Peptide Receptor Radionuclide Therapy) use has also been suggested for inoperable NETs (14).

The real-world effectiveness and tolerability of [^177^Lu]Lu-DOTATATE in patients with non-ileal primaries and in 3rd or later lines of treatment have not been well characterized.

## 2 Manuscript formatting

### 2.1 Materials and methods

### 2.2 Patients and therapy

This was a retrospective analysis of patients with histologically confirmed well-differentiated metastatic NETs who received [^177^Lu]Lu-DOTATATE. All patients had radiotracer uptake on [^68^Ga]Ga-DOTATATE PET/CT, defined as a lesion with Krenning score 3 or greater. Patients who received at least 1 cycle of [^177^Lu]Lu-DOTATATE were included in the analysis. Patients in this study were treated at a single tertiary-care institute.

During each cycle of [^177^Lu]Lu-DOTATATE, premedication with ondansetron 8 mg IV, dexamethasone 10 mg IV, and an amino acid infusion of L-Lysine 2.5%/L-Arginine 2.5% in 1 L normal saline were given 30 minutes before [^177^Lu]Lu-DOTATATE. [^177^Lu]Lu-DOTATATE (7.4GBq) was administered over 30 minutes. The amino acid infusion continued for a total of 4 hours. Treatment with [^177^Lu]Lu-DOTATATE was up to 4 cycles, given approximately every 8 weeks apart.

### 2.3 Follow-up

All patients treated with [^177^Lu]Lu-DOTATATE were clinically evaluated before each cycle. Evaluations included clinical assessment of symptoms, treatment toxicities, ECOG performance status, laboratory testing including complete blood counts with differential, serum electrolytes, renal function, hepatic function. Imaging with [^68^Ga]Ga-DOTATATE PET/CT or CT of the Chest/Abdomen/Pelvis with contrast was completed at approximately 3 month intervals.

### 2.4 End points and statistical analysis

Objective response rate (ORR) was evaluated according to RECIST 1.1 criteria. Complete response (CR) was defined as disappearance of all pathologic lesions. Partial response (PR) was defined as ≥ 30% decrease in the sum of the diameters from baseline sum diameters. Progressive disease (PD) was defined as ≥ 20% increase in the smallest sum of the diameters. Stable disease (SD) met none of the criteria above. Progression free survival (PFS) was measured as the time between the date of first [^177^Lu]Lu-DOTATATE infusion and progression of disease on imaging. Overall survival (OS) was measured from the date of first [^177^Lu]Lu-DOTATATE infusion to the date of death from any cause or date of last clinical follow-up. Patients alive at the last follow-up were censored on that date. Toxicity and adverse events were evaluated according to CTCAE v5.0 criteria. Continuous variables were summarized using the median. Categorical variables were summarized using basic proportions. Kaplan-Meier curves were used to depict survival.

### 2.5 Results

Of the 25 patients who were treated with [^177^Lu]Lu-DOTATATE, 13 (52%) were male, and 12 (48%) were female. The median age was 63 years (range 45 – 79). WHO Grade 1, 2, and 3 diseases were present in 5 (20%), 17 (68%), and 3 (12%) of patients, respectively. Fifteen patients (60%) received [^177^Lu]Lu-DOTATATE in the 2nd line setting, 5 patients (20%) in the 3rd line setting, and 4 patients (16%) in the 4th line setting. The most common primary sites of disease were the pancreas (40%) and the small bowel (40%). Only 4 patients (16%) had an ileal primary. The liver was the most common site of metastasis (92%), followed by bone (32%) (**Table 1**). The disease control rate was 92%, comprising 9 patients with a partial response (PR) and 14 patients with stable disease (SD). The objective response rate was 36%, comprised of the 9 patients who achieved PR. The median PFS was 28.5 months. Median OS was not reached. Two patients (8%) had progressive disease after starting treatment. The ORR in patients with non-ileal primary disease was 33.3%, and in patients with ileal primary disease was 50%. The ORR in patients with WHO Grade 1, 2, and 3 disease was 60%, 35%, and 0%, respectively. The ORR in patients who received [^177^Lu]Lu-DOTATATE in the 2nd line was 37.5%, 40% in the 3rd line, and 25% in the 4th line. The most frequently reported (>15%) nonhematologic treatment-related adverse events were fatigue, nausea, vomiting, and diarrhea. Clinically significant myelosuppression occurred in less than 5% of the patients. One patient experienced grade 3 fatigue and anorexia (**Table 2**).

**Table 1:** Demographics and baseline clinical characteristics of all patients.

Characteristic	Patients who received [^177^Lu]Lu-DOTATATE (%)
Sex:	
Female	13(52%)
Male	12(48%)
**Median age (years)**	63
Primary site of tumor:	
Pancreatic	10(40%)
Small Intestine, not otherwise specified	4(16%)
Ileum	4(16%)
Jejunum	1(4%)
Duodenum	1(4%)
Colorectal	3(12%)
Adrenal	1(4%)
Thymic origin	1(4%)
Sites of metastatsis:	
Liver	23(92%)
Bone	8(32%)
Mesentery	8(32%)
Spleen	2(8%)
Lung	2(8%)
Mediastinum	1(4%)
Peritoneum	1(4%)
WHO Grade:	
Grade 1	5(20%)
Grade 2	17(68%)
Grade 3	3(12%)

**Table 2:** Adverse events.

Event	Grade 1 N(%)	Grade 2 N(%)	Grade 3 N(%)
Gastrointestinal disorders:			
Nausea/vomiting	5(20%)	0	1(4%)
Diarrhea	5(20%)	1(4%)	0
Constipation	1(4%)	0	0
Hepatotoxicity	1(4%)	0	0
Hematologic disorders:			
Anemia	5(20%)	0	0
Thrombocytopenia	3	0	0
Pancytopenia	0	0	1(4%)
Neutropenia	0	1(4%)	0
Musculoskeletal			
Mucositis	1(4%)	0	0
Nail changes	2(8%)	0	0
Neuropathy	2(8%)	0	0
Other:			
Fatigue	14(56%)	1(4%)	1(4%)
Dehydration	0	0	1(4%)
Anorexia	3(12%)	1(4%)	1(4%)
Hypertension	2(8%)	1(4%)	0
Acute kidney injury	1(4%)	0	0
Insomnia	2(8%)	0	0

## 3 Discussion

The landmark study, NETTER 1, tested the efficacy of [^177^Lu]Lu-DOTATATE in combination with octreotide as opposed to octreotide alone in patients with histologically confirmed well-differentiated metastatic NETs originating in the midgut. It showed an improvement in median PFS of 40 months in patients who received the combination of octreotide plus [^177^Lu]Lu-DOTATATE as compared to 8.4 months in patients who received octreotide LAR alone. Though there was no statistical significance in median OS, there was a 11.7 month difference in the median OS which was considered clinically relevant (13).

Another retrospective study tested the same theory but in NETs originating from different organs including histologic grade 3 with high uptake on [^68^Ga]Ga-DOTATATE PET/computed tomography (CT) scans. They established an estimated median PFS of 36.4 months (about 3 years) and the mean OS was 61.9, 52.2, and 38.4 months (about 3 years) in WHO grades 1, 2, and 3 respectively. It was established that PRRT was beneficial in patients with SSTR2-positive pancreatic, non-pancreatic GEP NETs, lung NETs and metastatic NETs with an unknown primary site (15).

Currently, [^177^Lu]Lu-DOTATATE is the only approved PRRT for GEP NETs. Previous studies with other compounds in this category have been limited owing to significant hematologic and renal toxicities (16, 17).

Studies have shown that grade 3/4 hematologic toxicity in patients receiving PRRT has been in the acceptable range and patients with baseline poor renal function are more prone to this (18). As such, the incidence of PRRT-induced nephrotoxicity is low (19).

Disease progression can occur despite first-line therapy and hence retreatment with additional cycles has been suggested.

In a novel single-center study, it was shown that treatment with additional doses of [^177^Lu]Lu-DOTATATE, beyond the proposed 4 doses in patients with progressive NETs, was well tolerated and offered disease control (20).

A multicenter, randomized, controlled, open-label, phase 2 study called ReLUTH is currently ongoing in France which is aimed at testing the efficacy of retreatment with 2 cycles of Lutathera versus active surveillance for 6 months for patients with disease progression (21).

As evidenced by our study, [^177^Lu]Lu-DOTATATE demonstrated a notable response and showed activity among patients with the non-ileal and heavily pre-treated disease. Interestingly, response rates were worse in higher grade disease. Treatment was overall well tolerated, and the safety profile was consistent with published data. The role of tumor grade and non-ileal location for predicting response to [^177^Lu]Lu-DOTATATE merits further research.

## Data availability statement

The raw data supporting the conclusions of this article will be made available by the authors, without undue reservation.

## Ethics statement

The studies involving humans were approved by Allegheny Health Network Ethics Committee. The studies were conducted in accordance with the local legislation and institutional requirements. The participants provided their written informed consent to participate in this study.

## Author contributions

SI: Conceptualization, Investigation, Resources, Writing – original draft. EZ: Data curation, Formal analysis, Methodology, Validation, Writing – review & editing. DM: Supervision, Writing – review & editing. MR: Supervision, Writing – review & editing. NB: Supervision, Writing – review & editing.
