# Supplementary figures and images for: Long-term clinical outcomes of [177Lu]Lu-DOTATATE in patients with metastatic neuroendocrine tumors
Source: Front Oncol. 2024 May 16;14:1393317. doi: 10.3389/fonc.2024.1393317 (PMC11137281; doi:10.3389/fonc.2024.1393317)

## 1.1 Supplementary Figures

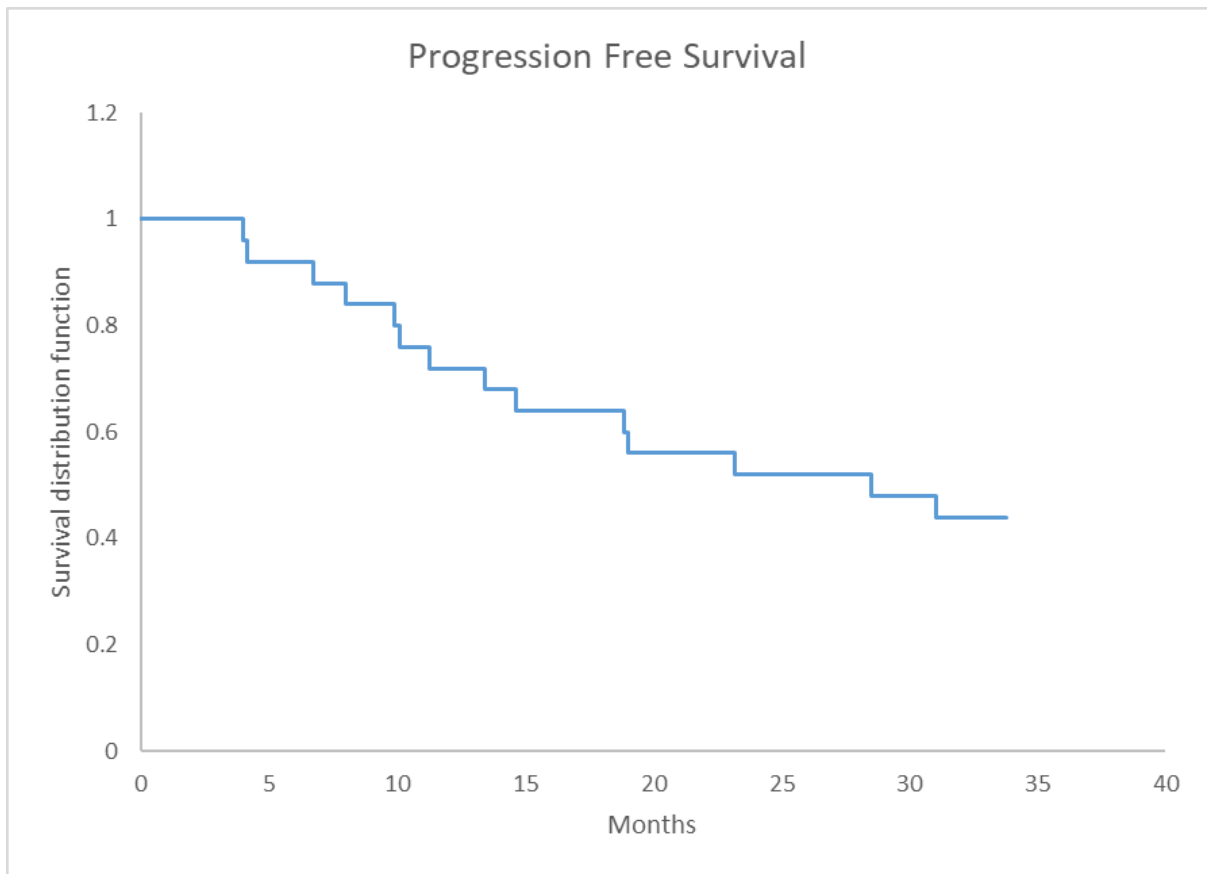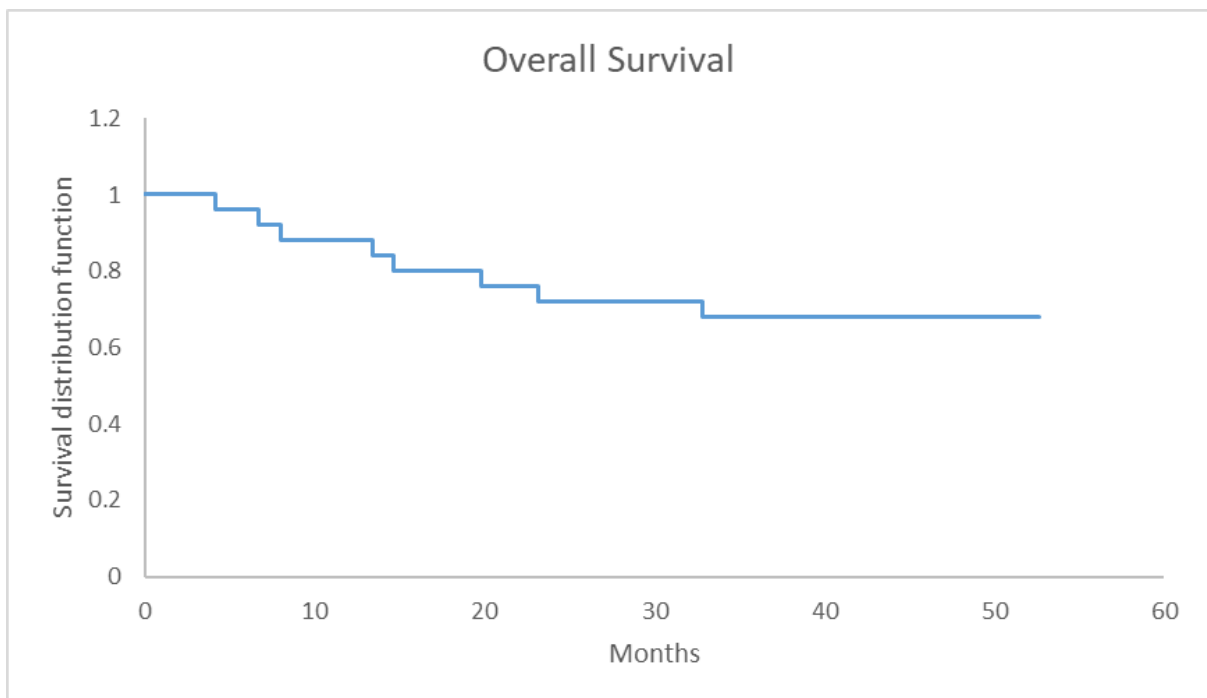

Supplement: Supplementary file 1 [file Image_1.pdf]
